# Impact of Continuing Care on Recovery From Substance Use Disorder

**DOI:** 10.35946/arcr.v41.1.01

**Published:** 2021-01-21

**Authors:** James R. McKay

**Affiliations:** Department of Psychiatry, University of Pennsylvania, Philadelphia, Pennsylvania. Corporal Michael J. Crescenz Veterans Affairs Medical Center, Philadelphia, Pennsylvania

**Keywords:** substance use disorder, treatment, continuing care, review, recovery, alcohol

## Abstract

Continuing care is widely believed to be an important component of effective treatment for substance use disorder, particularly for those individuals with greater problem severity. The purpose of this review was to examine the research literature on continuing care for alcohol and drug use disorders, including studies that addressed efficacy, moderators, mechanisms of action, and economic impact. This narrative review first considered findings from prior reviews (published through 2014), followed by a more detailed examination of studies published more recently. The review found that research has generally supported the efficacy of continuing care for both adolescents and adults, but the picture is complex. Reviews find relatively small effects when results from individual studies are combined. However, continuing care of longer duration that includes more active efforts to keep patients engaged may produce more consistently positive results. Moreover, patients at higher risk for relapse may benefit to a greater degree from continuing care. Several newer approaches for the provision of continuing care show promise. These include incentives for abstinence and automated mobile health interventions to augment more conventional counselor-delivered interventions. Primary care can be used to provide medications for opioid and alcohol use disorders over extended periods, although more research is needed to determine the optimal mix of behavioral treatments and other psychosocial services in this setting. Regardless of the intervention selected for use, the status of most patients will change and evolve over time, and interventions need to include provisions to assess patients on a regular basis and to change or adapt treatment when warranted.

As the substance use disorder (SUD) treatment system has evolved, the term “continuing care” has come to have two meanings.^1^–^4^ As originally conceptualized, continuing care was a period of lower-intensity treatment following a more intensive initial period, such as residential care or an intensive outpatient program (IOP).^2^,^4^ As such, continuing care was synonymous with “aftercare” or “stepdown care.” In this model, the goals of continuing care were to solidify and sustain the gains made in the initial phase of treatment, to establish abstinence if it was not already achieved, and to prevent subsequent relapses from worsening to the point that further acute treatment was necessary. In addition, disease management models of SUD treatment, sometimes delivered via primary care or via regular checkups, have attempted to improve outcomes by managing patients over extended periods. These models also can be seen as continuing care approaches.^1^,^3^

Due to the recognition that substance use disorder can be a chronic, long-term disorder, there has been an increase in research on how to improve the effectiveness of continuing care. The purpose of this review is to provide an update on the latest research on SUD continuing care, including newer approaches such as incentives, primary care–based clinical management, measurement-based care, adaptive treatment models, and mobile health components. The review begins with a brief summary of prior reviews (published through 2014) of SUD continuing care research. First, however, this review presents a conceptual model of continuing care and its principal goals with regard to the promotion of extended recovery.

## CONCEPTUAL MODEL

A return to substance use following a period of abstinence involves a number of distal and proximal factors, as outlined by Witkiewitz and Marlatt in their dynamic model of relapse.^5^ Factors such as family history of SUD, social support, self-efficacy, craving, and outcome expectancies account for level of general vulnerability to relapse. When high-risk situations are encountered, these factors—along with current affective state and the degree to which an effective coping behavior is performed—determine whether relapse occurs. Long-term recovery is a function of a number of factors, including characteristics of the individual’s relapse vulnerability as described in the Witkiewitz and Marlatt model, type and duration of treatment received including continuing care, and a variety of non-treatment factors experienced during and after formal treatment.^1^,^2^,^6^ These factors include participation in mutual help organizations, other forms of social support, and engagement in organizations and activities that promote recovery.

The important functions of continuing care in the recovery process involve maintaining abstinence/initial treatment gains; addressing relapse/non-response, including limiting the severity of relapses; connecting patients to other sources of support; and addressing other recovery issues, including employment, recreation, housing, and involvement in meaningful and/or enjoyable activities. Many of these functions are included in Wagner et al.’s chronic care model,^7^ which features interventions to increase self-confidence and skill levels, a focus on goal setting, identification of barriers to achieving goals, methods to overcome such barriers, support for patient self-management, and links to community resources.

Two important challenges faced during the continuing care phase of treatment are patient dropout and changes in the patient’s clinical needs over time. Therefore, effective clinical care must include elements that facilitate better retention and must be flexible enough to adapt to the changing needs of individuals. This review examines strategies that address these two issues, including active outreach to patients, use of incentives, measurement-based care, and adaptive treatment.

## METHODS USED IN THE REVIEW

PubMed and PsycINFO were used to identify prior reviews of the continuing care research literature as well as articles published after 2014 that were not included in these reviews. The search terms included substance use disorder, addiction, drug use disorder, alcohol use disorder, continuing care, aftercare, stepped-care, treatment outcome, efficacy, effectiveness, and cost-effectiveness. Studies without control groups were excluded from the review, with the exception of one study on the first evaluation of an intervention based on a package of services formerly offered only to pilots and doctors. Studies were not excluded for other methodological reasons or for country of origin.

## PRIOR REVIEWS OF CONTINUING CARE

### Adult Participants

One of the first reviews of continuing care included studies of continuing care versus no continuing care or minimal continuing care as well as studies comparing two or more active continuing care interventions.^2^ This review reported mixed results, with approximately half the interventions producing positive effects. Compared to studies with negative findings, the studies that generated positive effects tended to feature continuing care interventions with longer planned durations (at least 12 months), more active efforts to engage and retain patients, and weaker control conditions. A subsequent meta-analysis focused on 19 randomized trials published through 2010 that compared continuing care for SUD with minimal or no continuing care.^8^ The results of this study indicated a small but significant benefit for continuing care on SUD outcomes at the end of the interventions (*g* = .19, *p* < .001) and at post-treatment follow-up (*g* = .27, *p* < .01). (Hedges’ *g* and Cohen’s *d* are roughly equivalent measures of effect size.)

A systematic review of six methodologically rigorous trials of continuing care for alcohol use disorder found similarly mixed results.^9^ The trials tested multimodal interventions based on the chronic care model following initial treatment in more intense addiction and psychiatric programs. The interventions included a range of active outreach techniques, from telephone calls to follow-up by nurses, and various forms of individual or couples counseling. Four of the six trials found that patients receiving continuing care supplemented by active outreach interventions had significantly better drinking outcomes than patients receiving usual continuing care. In summary, prior reviews on the adult SUD continuing care literature found on average relatively small positive effects, which appeared to mask a fair amount of heterogeneity in results across studies.

### Adolescent Participants

Studies of continuing care for adolescents were reviewed by Passetti and colleagues.^10^ This review identified six studies with randomized designs, and four of these studies evaluated assertive continuing care (ACC).^11^ ACC consists of home visits, linkage to other services, transportation to services or other pro-recovery activities, advocacy to access services, and provision of the evidence-based adolescent community reinforcement approach (A-CRA).^12^ In three of the four studies of ACC, this intervention produced significantly better SUD outcomes than the continuing care provided as treatment as usual (TAU).^11^,^13^,^14^ A second intervention, active aftercare, whether delivered via in-person or telephone sessions, was found to be more effective than no aftercare (control condition).^15^ Finally, the effects of A-CRA versus continuing care with enhanced cognitive behavioral therapy (CBT) for adolescents who did not achieve abstinence in the initial phase of treatment were studied by Kaminer and colleagues.^16^ There were no differences in retention or abstinence rates between the two treatment conditions. It should be noted that three of these studies also were included in the review by Blodgett et al.^8^ In summary, prior reviews of continuing care for adolescents with SUD generally found favorable results, particularly for ACC.

## CONTINUING CARE STUDIES NOT INCLUDED IN PRIOR REVIEWS

A number of continuing care studies were not included in these reviews, primarily because they were published after 2010.

### Mindfulness-Based Relapse Prevention

Mindfulness-based relapse prevention (MBRP), an intervention that combines mindfulness practices and CBT relapse prevention (RP), was evaluated in a study by Bowen and colleagues.^17^ Participants who had successfully completed the first phase of treatment were randomly assigned to aftercare—MBRP, RP, or TAU (12-step programming and psychoeducation)—and followed for 12 months. Participants in MBRP and RP had lower rates of relapse to substance use and heavy drinking than did those in TAU. Moreover, among participants with some substance use, those in MBRP and RP had fewer days of substance use and heavy drinking than did those in TAU. RP was superior to MBRP in time to first drug use. Conversely, MBRP produced fewer days of reported substance use and heavy drinking at 12 months than did RP and TAU. These findings suggest that MBRP may be at least as effective as RP.

### Telephone-Based Continuing Care

#### Efficacy and effectiveness analyses

McKay and colleagues have published results from three additional telephone-based continuing care studies that were not included in earlier reviews.^2^,^8^,^9^ The first of these was conducted among participants with cocaine use disorder who had participated in an IOP for 2 to 4 weeks.^18^ About 40% of the sample also had current co-occurring alcohol use disorder (AUD). Participants were randomly assigned to IOP (TAU); IOP plus telephone monitoring and counseling (TMC), which consisted of up to 39 calls provided on a titrated schedule over 24 months; or IOP plus TMC with incentives for completed continuing care sessions (i.e., $10 gift coupons for each continuing care session attended in the first year), and followed for 24 months. The primary outcome was a composite measure that considered cocaine use, other drug use, and heavy alcohol use. There were no significant treatment main effects in this study. However, among participants who continued to use cocaine or drink alcohol in the first 3 weeks of IOP, TMC had significant positive effects on the primary outcome compared with TAU with IOP. Although the incentives almost doubled the number of continuing care sessions that were attended, substance use outcomes in the TMC plus incentives condition were slightly worse than those in TMC.

A second study, also focused on IOP patients with cocaine use disorder, evaluated an augmented version of TMC plus incentives for attendance that was provided to patients from the beginning of IOP, rather than only to those patients who had been attending IOP for several weeks.^19^ This 12-month version of TMC also included more vigorous outreach efforts when patients stopped completing calls, and more active efforts to link patients to recovery services in the community. Results of this randomized study indicated that this intervention actually produced worse results than the comparison condition, IOP only, over the 12-month follow-up, as indicated by the composite measure described above and cocaine urine toxicology. The authors speculated that providing such an intensive continuing care intervention in parallel with IOP may have overburdened and possibly confused patients in the study. Finally, 12-month outcomes from an ongoing study examining a 12-month version of TMC and a smartphone recovery program indicated that patients randomized to TMC had better outcomes on measures of status and frequency of alcohol use and heavy alcohol use than did those randomized to TAU.^20^

The impact of telephone continuing care on criminal justice outcomes was examined by combining patients with cocaine use disorder from three continuing care studies^8^,^21^,^22^ and comparing outcomes among those randomized to IOP plus TMC and those randomized to IOP only.^23^ The outcome measure was criminal convictions in the 4 years after admission to treatment. Controlling for a criminal sentence in the year prior to baseline, gender, age, and continuing care study, people with cocaine use disorder randomized to an IOP plus a telephone-based continuing care intervention had 54% lower odds of a criminal conviction and sentence in the 4 years after enrollment into the continuing care study, compared to those randomized to an IOP alone.

A 12-week version of the TMC protocol described in the studies above also was evaluated by Timko and colleagues.^24^ Patients (90% male) with co-occurring SUD and a psychiatric disorder who were receiving treatment in an inpatient psychiatric facility were randomized to receive 12 weeks of TMC or standard continuing care. Outcomes obtained for up to 12 months post–continuing care indicated that TMC did not improve substance use outcomes or increase attendance at self-help programs compared to standard care. The authors speculated that the intervention may have been too brief and not intensive enough to improve outcomes in what was already a fairly comprehensive program. In addition, work by McKay and colleagues has indicated that TMC may be more effective for women than for men.^25^,^26^

#### Economic analyses

Two investigations of the economic impact of TMC also have been published. The first study^27^ examined the 12-week version of TMC that was evaluated by McKay and colleagues.^21^ The study found that TMC was less expensive per client ($569) than treatment as usual aftercare with group counseling ($870) or than individual RP ($1,684). TMC also was more effective, with an abstinence rate of 57% compared to 47% for TAU. Thus, relative to TAU, TMC produced a highly favorable negative incremental cost-effectiveness ratio (−$1,400 per abstinent year). TMC also proved favorable under a benefit-cost perspective.

The second study^28^ examined the 24-month version of TMC evaluated by McKay and colleagues.^18^ The study evaluated the cost-effectiveness of TMC with and without incentives as a continuing care protocol for individuals with cocaine use disorder. Results suggest that, for the average client, TMC is a cost-effective strategy for reducing substance use, particularly if society is willing to pay more than $30 per day of abstinence. TMC plus incentives, on the other hand, was less cost-effective than TAU and was slightly less effective and more costly than TMC alone.

The results are reinforced by the societal cost analysis, which indicated that TMC generated the greatest reduction in societal costs overall ($1,564 on average). However, the TMC plus incentives condition had very high net savings ($2,138 from provider perspective, and $1,343 from societal perspective) for those patients who had a poor initial response to IOP as indicated by continued substance use. This finding illustrates that, from an economic perspective, it is advantageous to monitor substance use early in treatment and to tailor continuing care on the basis of whether initial abstinence is achieved. Continued substance use early in IOP could flag higher-risk individuals who are more likely to require more extensive and expensive interventions such as TMC plus incentives to achieve good outcomes over longer periods of time. The results of this study suggest that for such individuals, increased societal benefit will more than offset the added costs of the more expensive continuing care intervention.

#### Mediation effects

In the McKay et al. study, the positive effects of telephone continuing care relative to TAU (group counseling) over a 2-year follow-up were mediated by self-help involvement during continuing care as well as self-efficacy and commitment to abstinence 3 months after treatment.^21^ Scores on these measures were higher in the telephone condition relative to TAU, the measures predicted subsequent substance use outcomes, and analyses indicated significant mediation effects.^29^

#### Summary

Telephone continuing care appears to improve outcomes consistently for individuals with AUD. The findings for individuals with drug use disorders are more varied, with some studies generating no effects or even negative effects and others yielding positive effects in the full sample or in higher-risk subsamples. In addition, telephone continuing care has been found to be cost-effective and cost-beneficial compared to TAU, and to reduce the risk of criminal convictions in the 4 years following treatment intake.

### Recovery Management Checkups

#### Efficacy and effectiveness analyses

Recovery management checkups (RMC) is a continuing care intervention that provides individuals who have entered treatment for SUD with long-term monitoring of their substance use and active attempts to reengage them in treatment when needed.^30^–^33^ In RMC, an in-person clinical assessment is provided every 3 months by using standardized instruments as well as urine testing for substance use. When the clinical assessment indicates a need for active treatment, individuals are transferred to a linkage manager, who uses motivational interviewing techniques to help them recognize and acknowledge their resumption of substance use and need for additional treatment. Formal barriers to reentering treatment are discussed and addressed, and scheduling and transportation to treatment are arranged.

Three randomized trials comparing the RMC intervention with TAU have found positive effects on substance use outcomes.^30^–^33^ The first study in this series assigned 448 adults with chronic substance use to receive RMC plus standard treatment for 2 years or standard treatment alone.^30^,^32^ More than 90% of those randomized to RMC were seen at each quarterly assessment; these adults received the intervention if they were designated as in need of treatment, as indicated by “out of control” use in the prior 90 days. In intent-to-treat analyses, patients assigned to the RMC group, compared to those who received standard treatment alone, had fewer quarterly assessments in which they were found to be in need of SUD treatment. However, there were no significant differences between the two groups in substance-related problems per month or in total days of abstinence.

A second study randomly assigned 446 adults with chronic substance use disorder to receive RMC plus standard treatment for 4 years or standard treatment alone.^31^ In intent-to-treat analyses, patients assigned to the RMC group had fewer quarters in which they were found to be in need of SUD treatment, fewer substance-related problems per month, and more total days of abstinence (1,026 vs. 932 days) compared with patients in the control group who got assessments only.

A third trial randomly assigned 480 female offenders referred from incarceration to community-based SUD treatment to TAU versus TAU plus RMC provided for 3 years.^33^ Results indicated that RMC was beneficial for women who were not on probation. For example, among women not on probation, those who received RMC, compared with those who received TAU alone, were more likely to receive any days of SUD treatment (9% vs. 5%), less likely to engage in weekly alcohol and drug use (47% vs. 60%), and less likely to engage in any HIV-risk behavior (66% vs. 73%). Conversely, there were no significant positive effects for RMC in women on probation, possibly because they were already closely monitored.

#### Economic analyses

Cost-effectiveness was examined in the study in which 446 adults with chronic SUD were randomized to receive RMC for 4 years or quarterly assessments only.^31^ Over the 4-year trial, RMC cost on average $2,184 more than conducting quarterly assessments only. The incremental cost-effectiveness ratio for RMC was $23.38 per day abstinent and $59.51 per reduced problem related to excessive substance use. When additional costs to society were factored into the analysis, RMC was less costly and more effective than quarterly assessment only.^34^

#### Summary

RMC has consistently produced better substance use outcomes and quicker reentry into treatment during relapses than have assessments without intervention. Results also have indicated that RMC is a cost-effective and potentially cost-saving intervention.

### Continuing Care Based on Physician Health Programs

The model of continuing care used to treat physicians and pilots features intensive treatment initially, combined with extended continuing care for 5 years or more, and frequent random drug testing over that period. The active ingredients of the intervention are thought to be rapid detection of relapse to facilitate outreach, accountability, and social support. Several residential programs have developed continuing care interventions based on this model. One of these programs, My First Year of Recovery (MyFYR), was recently evaluated in a single-group observational study with no control group.^35^ MyFYR consists of random urine toxicology tests, recovery coaching, and a web-based application that links important individuals in the patient’s life (e.g., spouse, employer, other family members, provider) and supplies updates to these individuals on the patient’s urine testing compliance and results.

This evaluation found that patients who received MyFYR provided 70% of the scheduled urine samples over a 12-month period, for an average of 16.4 urine samples per patient.^35^ As determined by urine toxicology and client and family reports, 54% of the patients had some use of alcohol or drugs during the follow-up period. Of these relapsed patients, 71% were retained or re-engaged in MyFYR. Of these retained or re-engaged patients, 50% were able to re-establish abstinence for 2 months or more, as documented by multiple negative urine toxicology results. These results suggest that continuing care based on physician health programs also may be effective for individuals who are not motivated to participate in order to regain or maintain a professional license and a high-paying job. However, randomized studies with proper control conditions are needed before any conclusions are drawn about the effectiveness of this approach.

## CARE MANAGEMENT IN PRIMARY CARE

Clinical trials have been conducted to determine whether management of SUD, including ongoing continuing care, is feasible in primary care. Fiellin and colleagues randomized primary care patients with opioid use disorder to standard medical management with once-weekly dispensing of buprenorphine–naloxone, standard medical management with thrice-weekly dispensing, or enhanced medical management with thrice-weekly dispensing.^36^ All treatments were provided for 24 weeks. Results indicated that there were no differences between the three conditions on any of the primary substance use or retention measures.

In a second study, 563 patients with alcohol or drug use disorders who were completing medically supervised detoxification were randomly assigned to chronic care management for substance use disorder in primary care or to usual care for these disorders in primary care.^37^ The chronic care management intervention was delivered by an interdisciplinary team consisting of a nurse care manager, a social worker, an internist, and a psychiatrist with addiction expertise. At the 1-year follow-up, the chronic care management group and the control group did not differ on abstinence from heavy drinking, opioids, and stimulants (40% vs. 42%). There were no significant differences in other outcomes except fewer alcohol problems were reported by those with alcohol use disorder in the chronic care management group, a small effect of questionable clinical significance. Moreover, a follow-up analysis from this study also found no positive effects for subsets of patients in the chronic care management group with co-occurring major depression or post-traumatic stress disorder.^38^

A third clinical trial randomly assigned 82 women with a history of homelessness and alcohol use problems to a 6-month chronic care intervention or to usual care from primary care doctors without specialized training in alcohol interventions.^39^ The chronic care intervention consisted of brief intervention by a primary care doctor, referral to alcohol treatment services, and ongoing support from a case manager. Both conditions significantly reduced their alcohol consumption. There were no differences between the groups in reductions in drinking, housing stability, or mental or physical health.

In a fourth clinical trial, 163 patients with a DSM-IV diagnosis of alcohol dependence treated in primary care were randomly assigned to 26 weeks of alcohol care management or to referral for standard treatment in a specialty outpatient addiction treatment program.^40^ The care management program, which was provided in person and by phone, focused on the use of pharmacotherapy and psychosocial support. Compared with patients in the standard treatment group, patients receiving care management attended clinic visits more frequently, were more likely to receive naltrexone (12% vs. 66%), and had a smaller proportion of heavy drinking days per month. Overall abstinence did not differ between groups.

These studies generated little evidence on how to improve the treatment of patients with a drug or alcohol use disorder in primary care. However, offering alcohol care management to patients in primary care who have AUD does appear to be more effective than referring them to specialty care.

## USE OF MOBILE HEALTH TECHNOLOGY IN CONTINUING CARE

There are three potential roles for mobile health technology such as smartphone and texting programs in the delivery of continuing care. First, the technology could be used in conjunction with other behavioral interventions to provide automated support between therapy sessions and to convey information on a patient’s status back to the provider. For example, the A-CHESS (Addiction–Comprehensive Health Enhancement Support System) smartphone program has a number of supportive functions that can be accessed 24/7, including a chat room populated by others using the app, a library of materials on how to handle risky situations and other stressors, relaxation aids, and rapid connections to specified social supports.^41^ In addition, the app sends out daily and weekly assessments to patients using the system, and the patients’ responses are available in a dashboard that can be accessed by providers. The system also can be set to automatically send emails to providers when a patient reports worrisome information. Second, apps and SMS (short message service) could be used as stand-alone continuing care, perhaps for individuals who have limited access to more traditional clinic-based continuing care and for those further along in recovery. Finally, mobile heath can be an option for individuals who prefer virtual rather than in-person treatment.

So far, the apps and SMS programs that have been developed for individuals with SUD tend to fall into two main types.^42^ Several programs provide simplified versions of complex evidence-based behavioral interventions, such as CBT and the community reinforcement approach. These programs include CBT4CBT^43^ as well as reSET and reSET-O by Pear Therapeutics. Others, such as A-CHESS,^41^ do not attempt to provide manualized therapy interventions such as CBT to users. Rather, they have a range of other features designed to support recovery, such as self-monitoring, information on dealing with high-risk situations, tools for relaxation or distraction, and ways of connecting with peers or treatment providers. Most of these interventions have not been developed specifically for continuing care, but could potentially be used in that role. However, A-CHESS and two texting interventions were designed for the provision of continuing care.

In a controlled trial of A-CHESS, patients with alcohol use disorder (*N* = 349) who had completed residential treatment were randomized to receive A-CHESS for 8 months or standard continuing care only.^41^ The participants continued to use the A-CHESS system throughout the 8-month period during which it was provided. At 8 months, 70% of subjects were using A-CHESS at least weekly, compared to 92% at 1 month. Overall, participants used the system on 40% of the days they had access to it. Although frequency of reported alcohol use was low in both conditions during follow-up, patients receiving A-CHESS reported 49% fewer days with risky drinking in the prior 30 days at the 4-, 8-, and 12-month follow-up as compared to those in TAU. Rates of alcohol abstinence within the prior 30 days were higher in A-CHESS than in TAU at the 8-month follow-up (78% vs. 67%) and the 12-month follow-up (79% vs. 66%). A secondary analysis found that the positive effects of A-CHESS were mediated by increases in participation in outpatient treatment but not by increases in attendance at mutual health groups.^44^

A second trial of continuing care for patients with AUD found that providing A-CHESS, a smartphone, and a data plan for 12 months significantly reduced days of alcohol use and heavy alcohol use over that period relative to patients who did not receive A-CHESS.^20^ However, a condition that combined both A-CHESS and TMC in an integrated package did not produce superior alcohol use outcomes to A-CHESS or TMC alone.^20^

The efficacy of a recovery support program with mobile texting, called Educating and Supporting inQuisitive Youth in Recovery (ESQYIR), was evaluated by Gonzales and colleagues.^45^ The intervention consisted of 12 weeks of daily text messages about disease management, which included monitoring, feedback, reminders, education, and support. Monitoring texts were sent out every afternoon, along with feedback texts tailored on the basis of responses to the monitoring texts. In the study, 80 youths who had completed an initial phase of treatment were randomized to aftercare as usual (referral to self-help programs) or to ESQYIR. At 6- and 9-month post-aftercare follow-up, youths randomized to ESQYIR were less likely than those in TAU to test positive for their primary drug. They also reported significantly higher self-efficacy and were more likely to participate in recovery-oriented activities. Secondary analyses found that the positive effect of the intervention was mediated by increased involvement in pro-recovery activities other than Alcoholics Anonymous (AA) or Narcotics Anonymous (NA), but not by participation in AA or NA.^46^

A randomized study in Switzerland evaluated a continuing care intervention using text messaging to monitor self-selected drinking goals. The intervention also provided motivational text messages and telephone calls when participants failed to achieve goals or asked for support.^47^ Participants in the SMS condition responded to 88% of the SMS prompts, and 44% sent at least one request for help. Compared to standard continuing care, the intervention reduced the rate of at-risk drinking from 42% to 29%, a nonsignificant decrease.

Finally, Rose and colleagues developed an automated continuing care intervention that is delivered by telephone via interactive voice response (IVR).^48^ Participants call into the system once per day to report on 16 factors, including substance use, mood states, craving, self-efficacy, risk situations, sobriety support, substance-free recreation, and coping. When participants are judged to be at risk based on this assessment, tailored feedback is provided. Other features include CBT skills encouragement, coping skills review, and coping skills practice. Each month, participants also receive a personalized voice message from a counselor, which includes comments on progress and suggestions. The IVR system was evaluated in a study in which individuals with AUD who had completed 12 weeks of CBT were randomized to 4 months of the IVR system or of usual care, and followed for 12 months.^48^ Most primary analyses indicated no differences in drinking outcomes between the two conditions. However, a group x time interaction on drinking days per week favored the IVR condition. In addition, in participants who were abstinent at the end of the 12-week initial CBT intervention, outcomes on any drinking at the 2- and 4-month follow-up and any heavy drinking at the 4-month follow-up favored IVR over usual care.^48^ However, given the large number of analyses performed, these positive results should be interpreted cautiously.

Most of these studies testing continuing care with mobile health interventions have yielded positive effects on substance use outcomes. However, despite the initial promise of mobile health interventions, significant challenges remain in the provision of continuing care via mobile health apps and SMS. A recent systematic review found rapidly declining rates of smartphone use in most studies of interventions for mental health problems.^49^ This has sometimes been the case with mobile health interventions for addiction.^20^,^42^ Also, potential users must have access to a smartphone and data plan, or a telephone with SMS capabilities for texting-based interventions.

## INCENTIVES FOR ATTENDANCE AND ABSTINENCE

Several studies have examined the impact of providing incentives either for attendance at continuing care or for drug abstinence during continuing care. In one study, patients with cocaine use disorder who had completed 2 to 4 weeks of an IOP were randomized to receive additional individual CBT for 5 months (yes/no) and to receive monetary incentives for cocaine abstinence over 12 weeks (yes/no) in a 2 x 2 design.^50^ In the group that received both CBT and incentives for abstinence, participants were eligible for the incentives only if they were attending CBT sessions. Results over an 18-month follow-up found a significant positive main effect for abstinence incentives, and the best outcome was obtained in the group that received both incentives and CBT.^50^ Kirby and colleagues compared the standard 12-week contingency management for cocaine abstinence protocol with an extended 36-week protocol in methadone-maintained adults with cocaine use disorder, and found that the extended protocol produced significantly longer durations of continuous cocaine abstinence during weeks 1 through 24 and higher rates of cocaine-free urine samples during weeks 24 through 36.^51^ A third study examined the impact of providing $10 as an incentive for each continuing care session attended in the first year of a 2-year intervention for IOP patients with cocaine use disorder.^18^ The incentive almost doubled the number of continuing care sessions attended, but had no effect on cocaine use outcomes or on overall drug and alcohol use. Finally, Lash and colleagues found that adding social reinforcement of abstinence to an intervention that included attendance contracts and prompts improved aftercare attendance and abstinence outcomes compared to contracts and prompts only.^52^ These studies have found strong evidence of the efficacy of providing incentives for abstinence during continuing care. However, there is no evidence that providing incentives for continuing care attendance improves outcomes.

## ADAPTIVE TREATMENT AND CONTINUING CARE

There is a great deal of heterogeneity in how individuals respond to SUD treatment, including continuing care.^4^ Even with the most effective interventions, a significant percentage of patients will not exhibit a strongly positive response. Therefore, it is important to be able to adapt, or adjust, treatment when patients are not getting better.^53^ Moreover, there can be considerable heterogeneity within individuals in how their recovery is progressing over time. For example, a patient may do well in the first phase of treatment and in the first few months of continuing care, but then relapse and have a difficult time regaining abstinence. In a number of other areas in medicine—such as infectious diseases, hypertension, and cancer—algorithms have been developed to aid physicians in selecting optimal “plan B” treatments when the initial treatment offered does not work well.

In the treatment of SUD, less is known about how to best address heterogeneity of response between patients and within patients. However, some initial progress has been made. RMC addresses within-patient heterogeneity in response over extended periods of time by providing assessments every 3 months, with a protocol to transition individuals back into SUD treatment if they return to heavy alcohol or drug use.^30^–^33^ The research on TMC found that this extended intervention was most helpful for patients who did not do well in the first month of IOP, as evidenced by continued substance use,^18^ poor social support,^25^ or low motivation for recovery.^25^ Results over a 24-month follow-up period identified several subgroups for which adding TMC to IOP was particularly effective relative to IOP only: participants with poor social support, those with less motivation for recovery, and those with more prior treatment experiences.^25^ In addition, TMC was more beneficial for women participants than for male participants in two studies.^25^,^26^

One study with adolescents sought to determine the kind of continuing care that was best for those who had a poor response to outpatient treatment.^16^ Adolescents who did not achieve abstinence after 7 weeks of outpatient treatment were randomized to 10 weeks of individual CBT or A-CRA. Of these patients, 37% completed continuing care and 27% achieved abstinence. However, there were no differences in outcome between the two continuing care conditions.

These findings suggest that assessments conducted prior to and during continuing care provide data that can be used to improve outcomes by triggering changes to treatment.^4^,^54^ Ideally, these assessments should address recent or current substance use as well as other factors that are linked to relapse. For example, current depression, craving poor social support, and lack of commitment to abstinence all have predicted subsequent relapse in multiple studies. Even if a patient remains abstinent during continuing care, it may be important to modify the intervention in some way if craving or depression increases.^4^

## RESEARCH ASSESSMENT EFFECTS

There is evidence that research follow-up can have a positive effect on alcohol and drug use outcomes in treatment studies. Clifford and colleagues found that study participants who received more follow-ups had significantly better alcohol use outcomes.^55^ In a second study, participants were randomly assigned to one of four research assessment follow-up schedules that varied by frequency and duration. Those assigned to the infrequent and brief assessment condition had worse drinking outcomes (i.e., higher frequency, greater quantity), higher negative consequences of drinking, and worse drug use outcomes than did those assigned to more frequent and longer assessments.^56^ Other studies in this area have produced more mixed results.^57^ Although the mechanisms of action are not well understood, the process of being asked about substance use may increase its salience for the participant, or may be therapeutic in some other way.

## MEDICATIONS

The U.S. Food and Drug Administration (FDA) has approved several medications for AUD and opiate use disorder. With regard to medications for AUD, there is no convincing evidence to date that longer periods of use produce better drinking outcomes than do shorter periods, or that using the medications in the context of continuing care produces better outcomes. However, this is largely because little research in this area has been done; most studies have evaluated only 12- or 24-week courses of medication. In one exception to this general trend, a study with male veterans with chronic, severe alcohol addiction found no differences between placebo, naltrexone for 3 months, and naltrexone for 12 months conditions in frequency of drinking or number of drinks per drinking day at 1-year follow-up.^58^ Conversely, there is good evidence that longer periods on medications for opiate use disorder produce better outcomes than shorter periods, and at this point, detoxification is not recommended.^59^ There are no FDA-approved medications for stimulant or cannabis use disorder. More research is needed to determine if longer durations on medications for AUD are beneficial, and to identify successful strategies to increase long-term use of effective medications.

## CONCLUSIONS

At this point, continuing care is widely believed to be an important component of effective treatment for substance use disorder, particularly for those individuals with a problem severe enough to require specialty care treatment. The research base generally has supported the efficacy of continuing care for both adolescents and adults, but the picture is complex. Reviews have found relatively small to moderate effects when results from individual studies are averaged or combined in some way.^2^,^8^ However, there is some evidence that continuing care of longer duration that includes more active efforts to keep patients engaged may produce more consistently positive results.^2^,^13^ Moreover, patients at higher risk for relapse—by virtue of continued substance use in the first phase of care, or poor social support or low motivation early in treatment—may benefit to a greater degree from continuing care than those patients with a better prognosis.^18^,^25^,^26^

Several new approaches show promise for the provision of continuing care. These include incentives for abstinence; use of automated mobile health interventions to augment more conventional counselor-delivered interventions; and extended treatment and monitoring programs that, until very recently, have been provided only to pilots and doctors. There is also evidence that primary care can be used to provide medications for opioid and alcohol use disorders over extended periods; however, more research is needed to determine the optimal mix of behavioral treatments and other psychosocial services in this setting. Regardless of the intervention selected for use, it is clear that the status of most patients with SUD will change and evolve over time, and interventions need to include provisions to assess patients on a regular basis and to change or adapt treatment when warranted.^4^,^25^,^26^,^54^ More research is needed to develop evidence-based protocols for adapting continuing care interventions over time and addressing nonresponse. In addition, to promote higher rates of stable, long-term recovery, additional work is needed to develop methods to integrate continuing care interventions more effectively with other supports available in the community and to promote greater involvement in rewarding activities that provide enjoyment and a sense of meaning and purpose.^6^

The field is also starting to move toward more specific guidelines regarding the characteristics of high-quality continuing care. A recent review of evidence-based guidelines and quality indicators derived 13 specific quality indicators, including the provision of information on self-help, relapse prevention strategies, involvement of family members, provision of both behavioral interventions and medications, minimum of 3 months of follow-up, and patient involvement in development of continuing care plans.^60^ The development of evidence-based clinical practice guidelines to facilitate wider implementation of effective continuing care would be a major advance for the field. As discussed here, these guidelines likely will need to include information on adapting continuing care over time at the individual level to achieve optimal outcomes. For example, higher-risk patients likely will benefit from continuing care interventions with longer durations, and some patients may have preferences for particular approaches or modalities (e.g., mobile health vs. clinic-based care).

Finally, although the efficacy of specific continuing care interventions is certainly important, the crucial roles played by providers who deliver these interventions have not received sufficient attention. Some providers are simply better than others, but the individual characteristics and training that facilitate greater success as a continuing care provider have received little attention. Intriguing work in this area has been done by Karno and Longabaugh, who conducted an elegant series of studies on the impact of continuing care therapist counseling style, and the interaction between counseling style and patient characteristics, on drinking outcomes.^61^,^62^ This work has involved the careful coding of therapist and patient behaviors during continuing care treatment sessions for factors such as focus on emotional material and directness.

In one study, patients with clinically elevated depression scores had better drinking outcomes if their therapists had a *low* focus on painful emotional material, and worse outcomes when the therapist was more focused on such material.^61^ Therapist focus on emotional material did not predict drinking outcomes in patients who were not depressed. A second study looked at therapist directiveness, or the degree to which the therapist employed confrontation, interpretation, and closed-ended questions; addressed in-session resistance; initiated topics; and provided information.^62^ Results indicated that higher therapist directiveness predicted worse drinking outcomes in high-anger patients, and better drinking outcomes in low-anger patients. Therefore, in addition to proceeding with the further development and evaluation of innovative continuing care interventions and methods of intervention delivery, much more attention should be devoted to improving the therapeutic skills of providers and studying the process of change within continuing care sessions.
